# Standard method for performing positron emission particle tracking (PEPT) measurements of froth flotation at PEPT Cape Town

**DOI:** 10.1016/j.mex.2022.101680

**Published:** 2022-03-26

**Authors:** Katie Cole, Daniel J. Barker, Pablo R. Brito-Parada, Andy Buffler, Kathryn Hadler, Isobel Mackay, Diego Mesa, Angus J. Morrison, Stephen Neethling, Alexander Norori-McCormac, Barry Shean, Jan Cilliers

**Affiliations:** 1 Department of Physics, University of Cape Town, South Africa; 2 Department of Earth Science and Engineering, Imperial College London, UK; 3 Department of Chemical Engineering, University College London, UK; 4 Julius Kruttschnitt Mineral Research Centre, University of Queensland, Australia; 5 Heron Resources, Australia

**Keywords:** PEPT, Particle tracking, Froth flotation

## Abstract

Positron emission particle tracking (PEPT) is a technique for measuring the motion of tracer particles in systems of flow such as mineral froth flotation. An advantage of PEPT is that tracer particles with different physical properties can be tracked in the same experimental system, which allows detailed studies of the relative behaviour of different particle classes in flotation. This work describes the standard operating protocol developed for PEPT experiments in a flotation vessel at PEPT Cape Town in South Africa. A continuously overflowing vessel with constant air recovery enables several hours of data acquisition at steady state flow and consistent flotation conditions. Tracer particles are fabricated with different coatings to mimic mineral surface hydrophobicity and size, and a data treatment derived from a rotating disk study is utilized to produce high frequency (1 kHz) location data relative to the tracer activity. Time averaging methods are used to represent the Eulerian flow field and occupancy of the tracer behaviour based on voxel schemes in different co-ordinate systems. The average velocity of the flow in each voxel is calculated as the peak of the probability density function to represent the peak of asymmetrical or multimodal distributions.

• A continuously overflowing flotation vessel was developed for extended data acquisition at steady state flow.
• The data treatment enabled the direct comparison of different particle classes in the flotation vessel.
• The solids flow fields was described by the probability density function of tracer particle velocity measured in different voxel schemes.

## Graphical abstract

**Figure:**
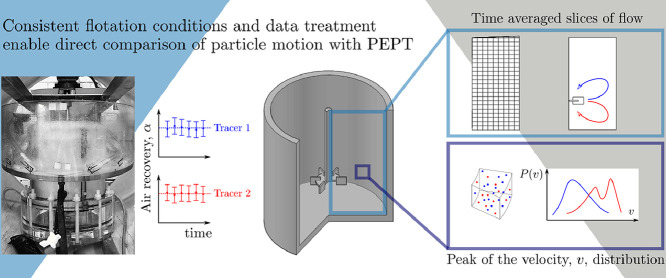

Specifications table

**Table:** 

Subject Area:	Chemical Engineering
More specific subject area:	Froth flotation
Method name:	PEPT measurements for froth flotation
Name and reference of original method:	A. Norori-McCormac, P. Brito-Parada, K. Hadler, K. Cole, J. Cilliers, The effect of particle size distribution on froth stability in flotation, Separation and Purification Technology 184 (2017) 240–247. D. Parker, C. Broadbent, P. Fowles, M. Hawkesworth, P. McNeil, Positron emission particle tracking - a technique for studying flow within engineering equipment, Nuclear Instruments and Methods in Physics Research Section A: Accelerators, Spectrometers, Detectors and Associated Equipment 326 (1993) 592 – 607. T. Volkwyn, A. Buffler, I. Govender, J.-P. Franzidis, A. Morrison, A. Odo, N. van der Meulen, C. Vermeulen, Studies of the effect of tracer activity on time-averaged positron emission particle tracking measurements on tumbling mills at PEPT Cape Town, Minerals Engineering 24 (2011) 261–266.
Resource availability:	*N/A*

## Introduction

Positron emission particle tracking (PEPT) is a radiation-based technique for measuring the location of a tracer particle with time within a system of interest [14]. It has grown to be well established within science and engineering research, particularly in the mineral separation process of froth flotation [11,17], for which the application of optical measurement techniques is impaired by the high density of the pulp phase and the opaque nature of the mineral-laden bubbles. PEPT location data can be used to describe the Lagrangian trajectory of the tracer particle and to calculate Eulerian descriptions of the flow such as velocity and acceleration fields as well as occupancy and residence times. These are valuable parameters to improve understanding of particle behaviour in complex and turbulent multi-phase systems such as flotation, and for the validation of theoretical models of flow.

Another major advantage of PEPT for flotation research is that it can be used to investigate the effect of particle properties, such as size, shape, surface hydrophobicity and density (e.g. [1,^6^,^7^,11]). The tracer particle for PEPT measurements is both labelled with a positron-emitting radionuclide and has physical and chemical properties that represent those of the flotation bulk. As the tracer particle moves around the interior of an experimental vessel, positrons released in the decay of the radionuclide from the tracer annihilate with local electrons to produce pairs of almost back-to-back 511 keV gamma rays. If both gamma rays in any pair are detected in coincidence within a positron emission tomography (PET) camera, a virtual line can be formed to indicate the path along which the annihilation event occurred. This is known as a line of response (LOR), and with many LORs, the 3D position of the tracer can be located; (X,Y,Z) in Cartesian co-ordinates. When a tracer particle is moving, the LORs can be analysed in chronological sets to derive the tracer location, which contains the tracer position at a specific time. The most widely used algorithm to find the tracer location, “*track”*, was developed by the University of Birmingham [14]. In more recent times, new location techniques have been developed for PEPT as reviewed by Windows-Yule et al. [18].

The protocol presented here was developed in collaboration between Imperial College London and PEPT Cape Town at the University of Cape Town to enable PEPT measurements in a flotation vessel.

## The standard method

The method consists of a series of sub-steps: flotation methods, PET scanner, tracer particles, PEPT measurement, protocol to locate freely moving particles based on a rotating disk study, calculation of velocity data, co-ordinate systems and time averaging methods.

### Flotation methods

**Fig. 1:**
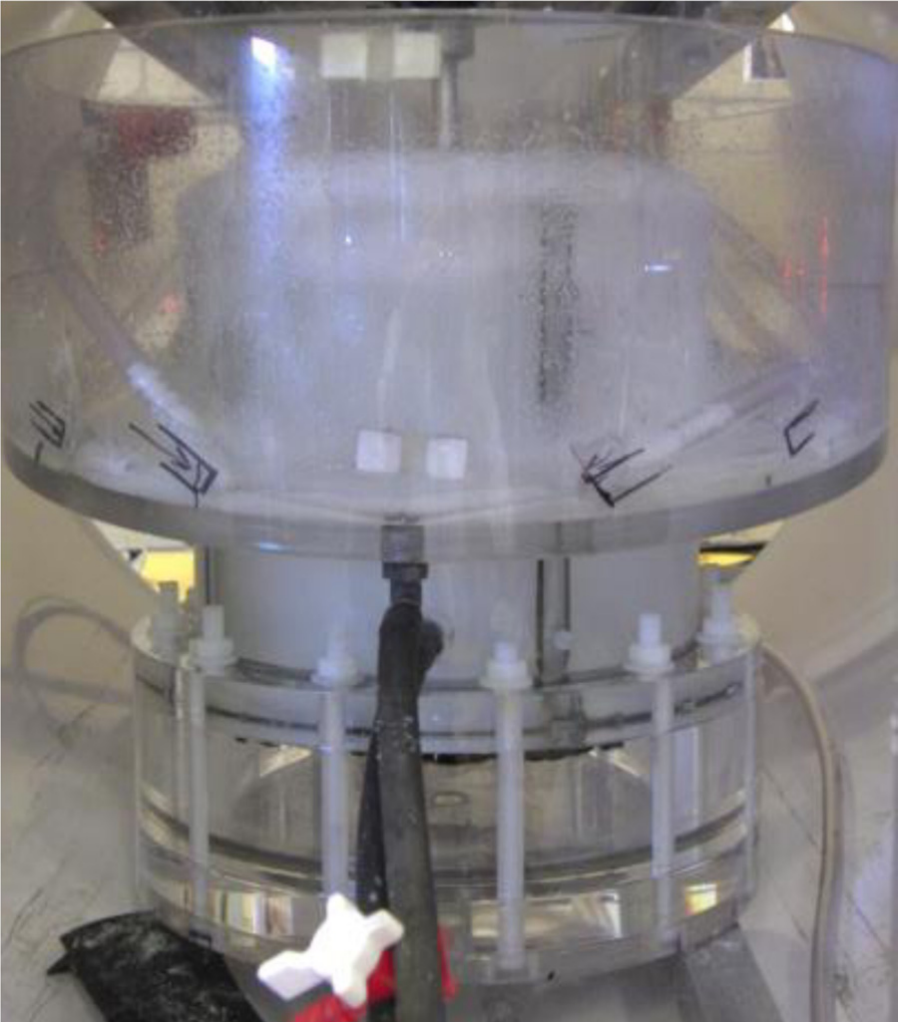
The standard flotation vessel with external launder.

The flotation vessel used for PEPT experiments is cylindrical and formed of acrylic (Stanley Plastics Ltd., UK) as described in Norori-McCormac et al. [13] and is shown in Fig. 1. The geometry is represented by a standard configuration, with the diameter and height both equalling 180 mm. The vessel contains four baffles with width one-tenth of the vessel diameter, spaced at intervals of 90° and at a height of two thirds of the vessel height. It is agitated with a six flat-bladed Rushton turbine of diameter one-third of the vessel diameter (60 mm), with width and height as one-quarter and one-fifth of the diameter, respectively. The impeller is driven with a frequency of 1200 rpm and resulting impeller tip speed of approximately 3.7 m s ^−^ ^1^.

Aeration is provided through a small air reservoir at the base of the vessel. It passes through a frit plate to disperse fine air bubbles, which is in the shape of a disk and fabricated from layers of sintered fine steel mesh with diameter 180 mm and mesh hole size 20 µm (Carbis Filtration Ltd., UK). The vessel is fitted with an external launder to enable continuous operation, with any overflowing material collected and pumped to the base of the vessel. It contains an air blow down system at 100 litres per minute to break down any froth and direct solids to the recycling pump feed. A peristaltic pump recycles slurry from the launder and returns it to the base of the vessel, at a height of 45 mm above the air plate. The recycle feed is directed towards the impeller via a hose barb on the inside of the vessel so that the returned material does not short circuit directly to the launder. Recycling the overflowed solids enables data collection over several hours with the same solid concentration in the pulp.

**Fig. 2:**
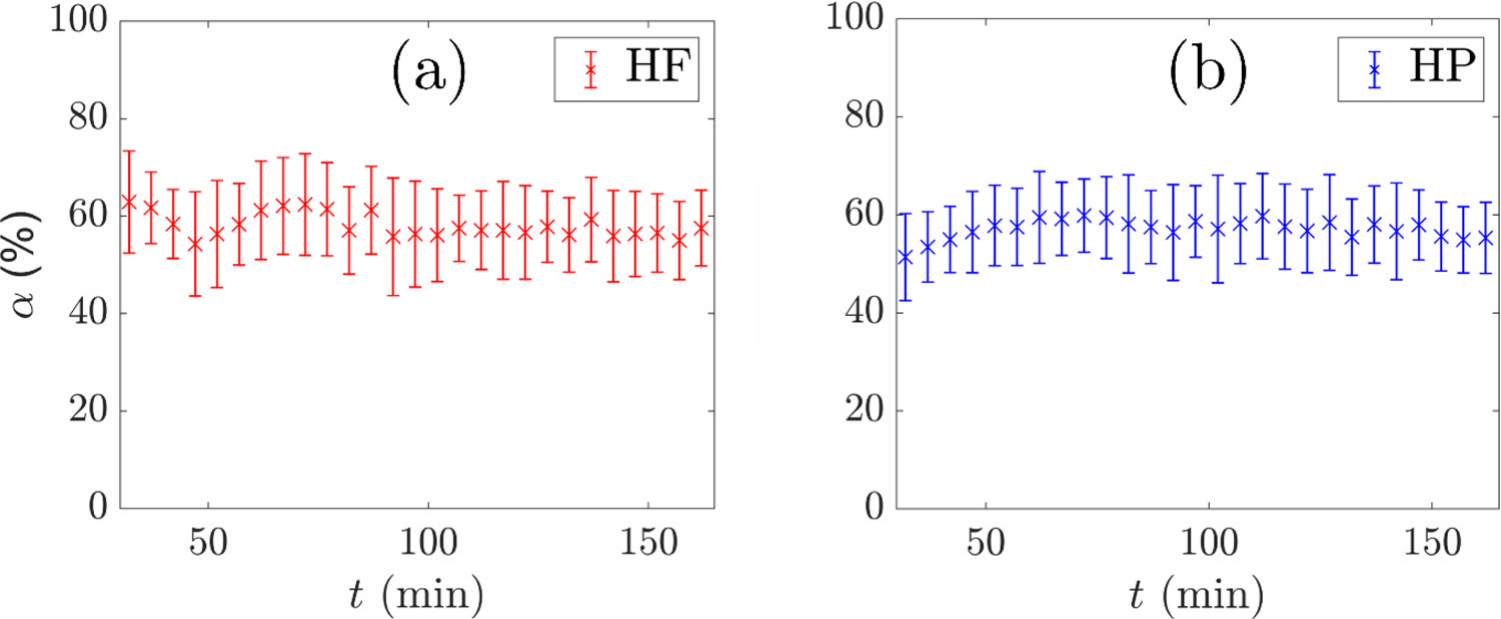
Air recovery, *α*, with time, *t*, in two separate flotation experiments with either a hydrophilic (HF) or hydrophobic (HP) tracer particle. The values represent the mean air recovery and standard deviation of samples taken every 5 minutes.

Online measurements of the overflowing froth height and velocity are performed as described by Norori-McCormac et al. [13]. A video camera and optical level sensor are placed over the lip to capture the velocity and height of the overflowing froth. These variables are monitored throughout each experiment to maintain consistent flotation behaviour during the experiment in terms of the air recovery, which is a parameter related to performance in mineral froth flotation [9,12], and has the advantage of being non-invasive. This enables repeat experiments with the same flotation conditions. Fig. 2 shows examples of consistent air recovery over the duration of two experiments with different tracer particles.

The air recovery measurements are used to determine when the vessel achieves steady state by the properties of the overflowing froth. The ambient temperature and temperature inside the field of view of the camera are monitored during experiments. The air recovery measurements are used to guide the additional dosing of volatile flotation reagents to enable longer experiments at consistent flotation performance.

### Positron camera

**Fig. 3:**
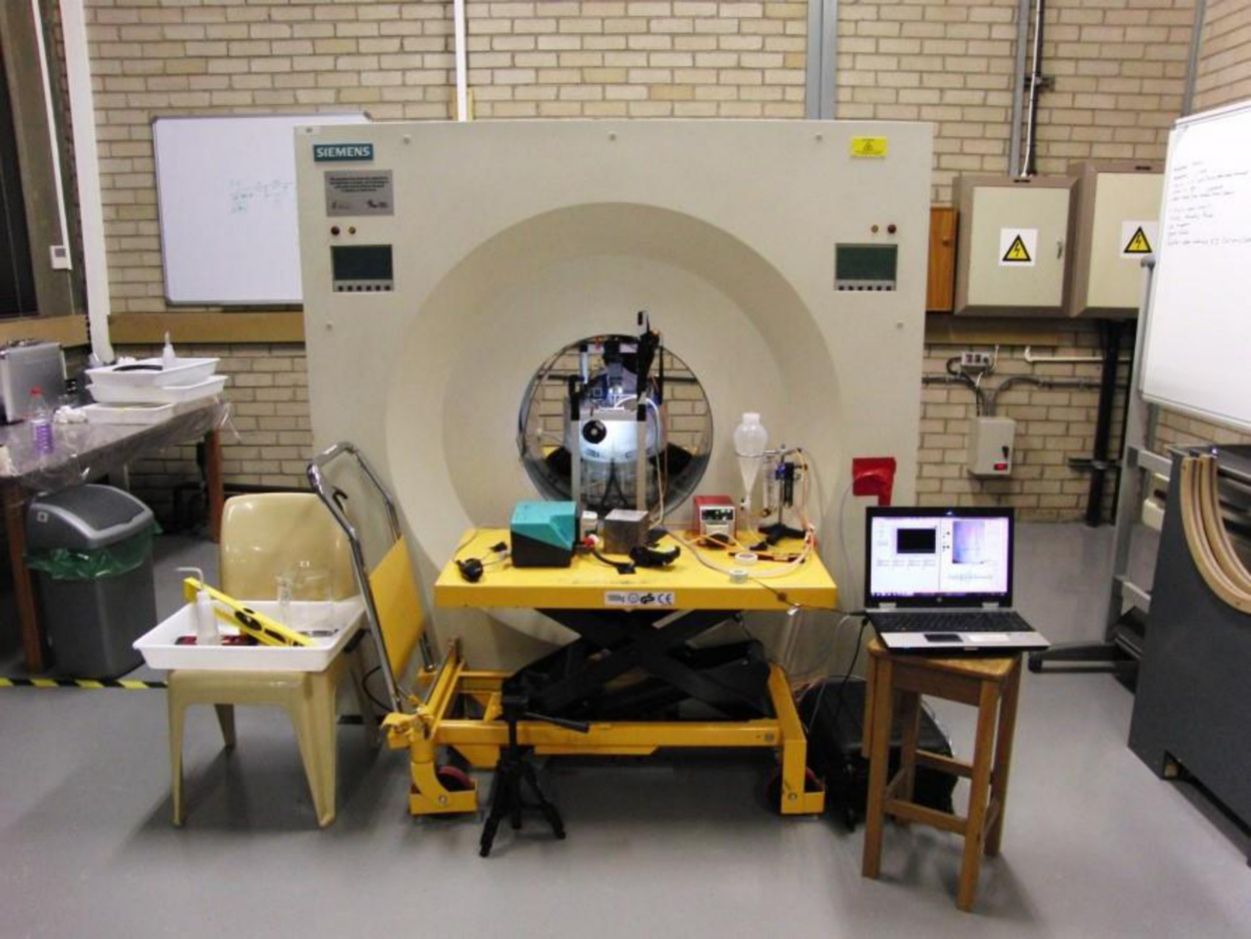
Experimental set-up showing the flotation vessel inside the HR++ scanner for PEPT experiments.

The PEPT experiments are performed at the laboratories of PEPT Cape Town, which houses an ECAT `EXACT3D' HR++ (Model: CTI/Siemens 966) PET camera [2]. The vessel is installed in the centre of the field of view of the positron camera via two truck trolleys and a supporting frame as shown in Fig. 3.

Specific aspects of the vessel were designed to enable PEPT experiments with the HR++ camera, with the vessel diameter and height designed to use the full axial length of the field of view of the camera. The impeller is mounted on the top of the vessel with a right-angled gearbox (Automation International Ltd., UK) to drive the impeller from outside the field of view of the positron camera. The use of steel and other dense construction materials is minimized, with the vessel material and fasteners fabricated from low density plastics such as acrylic to minimize the attenuation of gamma rays detected by the PET camera. The air frit plate is constructed from layers of fine sintered steel meshes to reduce the overall density while maintaining structural strength and to prevent deformation due to the upwards air flow.

### Tracer particles

Consistent vessel performance between repeat experiments enables PEPT measurements of different tracer particles under the same flotation conditions. Tracer particles for flotation studies are fabricated from a core of Purolite NRW100 ion exchange resin, by the method described in Cole et al. [5]. The core sizes start from approximately 350 µm which makes the tracer methods appropriate to study coarse particle flotation with PEPT. These particles can be radiolabelled with an initial activity of up to 1.5 mCi (56 MBq) of the PET radionuclide ^68^Ga which decays via positron emission with a half-life of 68 min. The initial radiolabeled activities are measured immediately after fabrication with an ionization chamber (model Capintec CRC-25R).

In flotation, surface hydrophobicity and size are two key properties related to performance. Tracer particles for PEPT are modified to represent different flotation particles. The native surface of the resin material is hydrophilic and so can be used as a hydrophilic tracer after labelling. For a silica-based flotation species, the resin particle can be coated with silanised silica (0 < *d*_silica_ < 50 µm) to create a hydrophobic tracer following the method of Cole et al. [6]. In this case, epoxy resin adhesive is used to fix the silica to the surface of the resin, so the coating can withstand the torturous environment near the impeller and in the peristaltic pump used to recycle overflowing material. The coatings lead to a difference in size and mass between tracer particles. To manage particle size, the core sizes and coating thickness are measured throughout the coating process by microscope image analysis and comparison with monodisperse particle size standards of diameter 98.1 ± 2.8 µm (Whitehouse Scientific Ltd., NIST Standard).

### PEPT measurements

PEPT measurements are performed by recording list-mode data in increments of up to 20 min, based on the maximum file size that can be handled by the data acquisition system. The list-mode data contain a list of lines of response recorded as pairs of positions in 3D of the two detector elements from each coincidence event and a time stamp every 1 ms. The PEPT location data are derived from the list-mode data using the University of Birmingham “*track”* algorithm [14] which is an iterative method based on slicing the lines of response into bins of size N and finding the closest passing fraction, f. The resulting location data are a series of positions, $\vec{P}\left(X,Y,Z\right)$, with time, t, where the co-ordinates X and Z represent the horizontal dimensions of the measurement, and Y corresponds to the vertical dimension which is offset to have the impeller at Y = 0 mm or a fractional height, Y/H = 0.33, where H is the total height.

In this protocol, the choice of parameters for locating a tracer particle are guided by a preliminary measurement with a tracer particle attached to the tip of an impeller blade in the flotation vessel. By using the movement of a tracer particle on a predictable path, it is possible to determine values of N andf to create location data with specific properties. In particular, an optimum value of f can be derived that minimises the statistical uncertainty in the location measurement, and N can be varied with activity to maintain a constant location rate in the data. This standard protocol follows the methods presented in Parker et al. [14] and Volkwyn et al. [16].

### Rotating disc study

Consider the results from a rotating disk study with a coarse tracer particle of diameter 500 µm radiolabeled with 660 µCi (24.4 MBq) of ^68^Ga by ion exchange [5]. The tracer particle was attached to the tip of the impeller in the flotation vessel. The vessel contains attenuating materials, such as stainless steel, acrylic and nylon plastics. List-mode data were acquired over four half-lives of ^68^Ga ($t_{1/2}$ = 68 min) and location data were determined with the “*track”* algorithm for a range of different bin sizes, N, and final fraction, f, values, to evaluate the statistical uncertainties of the PEPT measurement as the activity of the tracer decreased.

The X and Z co-ordinates of each PEPT measurement were considered as they represent the transaxial and axial dimensions of the field of view of the HR++ camera and are associated with different geometrical detection efficiencies, and the Y co-ordinate was fixed during experiments.

#### Methods to evaluate the standard uncertainty in the location data

Each co-ordinate of the tracer position with time, t, was predicted by the equations,

(1) $$\begin{array}{c} X_{fit}\left(t\right)=X_{0}+a\cos\left(2\pi \nu t+\phi \right)\;\;\text{and}\\ Z_{fit}\left(t\right)=\;Z_{0}+a\cos\left(2\pi \nu t+\phi +\frac{\pi }{2}\right), \end{array}$$

where a is the amplitude of the motion and the radius of the impeller, ν is the frequency of rotation of the impeller, for a phase angle, ϕ, and center of rotation (*Z_0_, X_0_*). An unconstrained nonlinear optimization was used to find the optimum values of a, ν, ϕ and (*Z_0_, X_0_*) for each combination of *N* and *f* based on an initial guess of the values from equipment settings. The root mean squared differences between the predicted and measured co-ordinates in each dimension with PEPT were calculated as,

(2) $$\begin{array}{ccc} \delta X & = & \sqrt{\frac{\sum_{i}^{n}{\left(\Delta X_{i}\right)}^{2}}{n}}\;\;\text{and}\\ \delta Z & = & \sqrt{\frac{\sum_{i}^{n}{\left(\Delta Z_{i}\right)}^{2}}{n}}, \end{array}$$

where $\Delta Z_{i}=X_{i}-X_{fit}\left(t_{i}\right)$ and $\Delta Z_{i}=Z_{i}-Z_{fit}\left(t_{i}\right)$ for each location i in a data set of size n = 1000 locations*.*

The standard uncertainties for each measured co-ordinate of the tracer position were calculated as,

(3) $$\begin{array}{c} u\left(X\right)=\frac{\delta X}{\sqrt{n}}\;\;\text{and}\\ u\left(Z\right)=\frac{\delta Z}{\sqrt{n}}\;. \end{array}$$

**Fig. 4:**
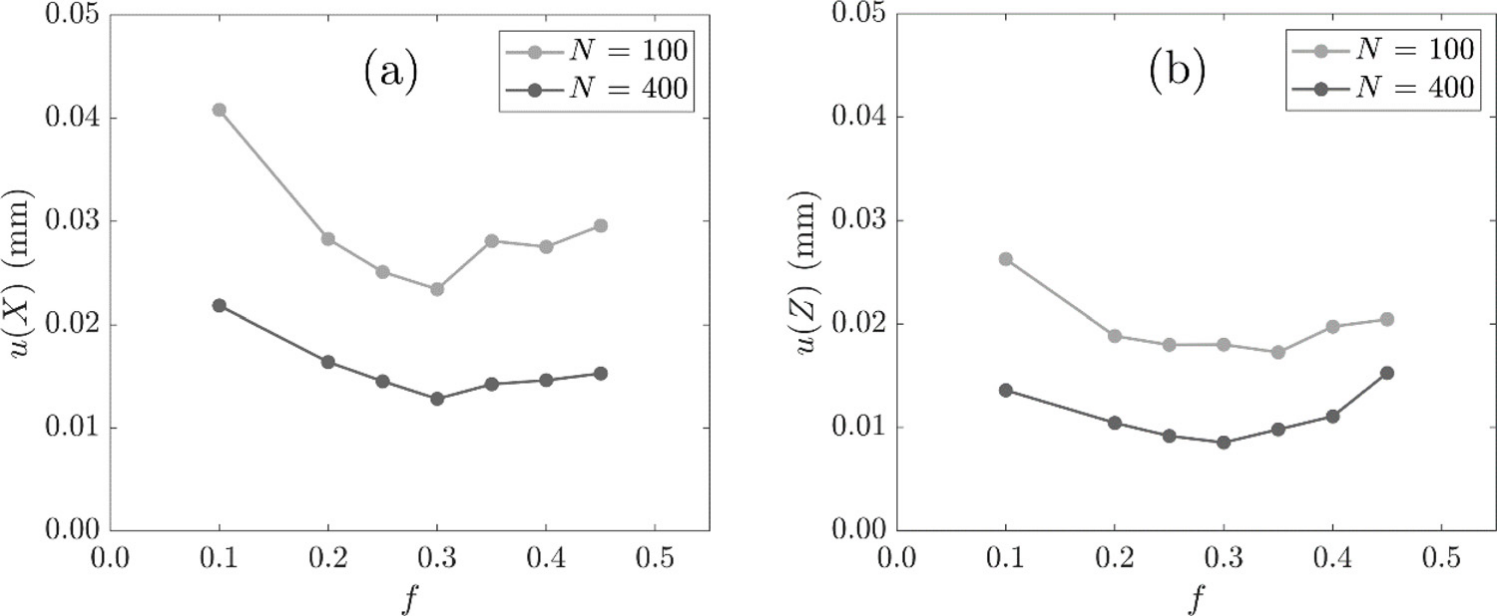
Uncertainty in the co-ordinates (a) X and (b) Z of the measured tracer position with f for two values of N and a fixed tracer activity of 520 µCi.

Fig. 4 shows the location uncertainty in two dimensions, X and Z, for a range of f and two values of N. The optimum fraction of events $f_{opt}$ was selected as 0.30, corresponding to the minimum location uncertainty at both N values considered and in both dimensions.

#### Methods to maintain a high location rate

**Fig. 5:**
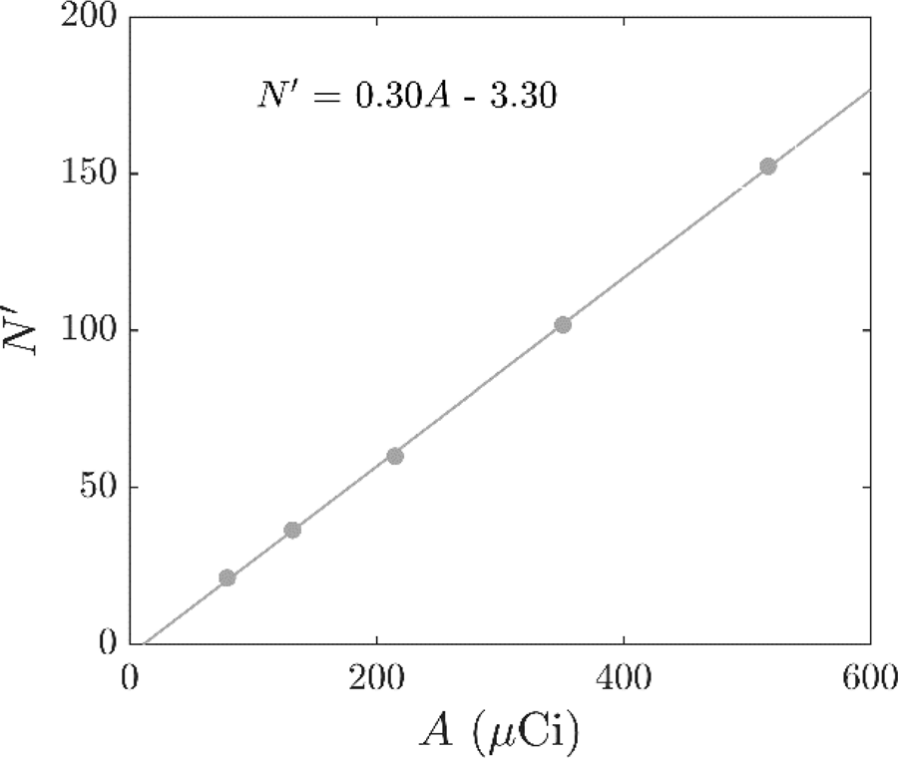
Target bin size $N^{\prime }$ against tracer activity A at f = 0.30. A linear fit with *R^2^* > 0.99 is included.

**Fig. 6:**
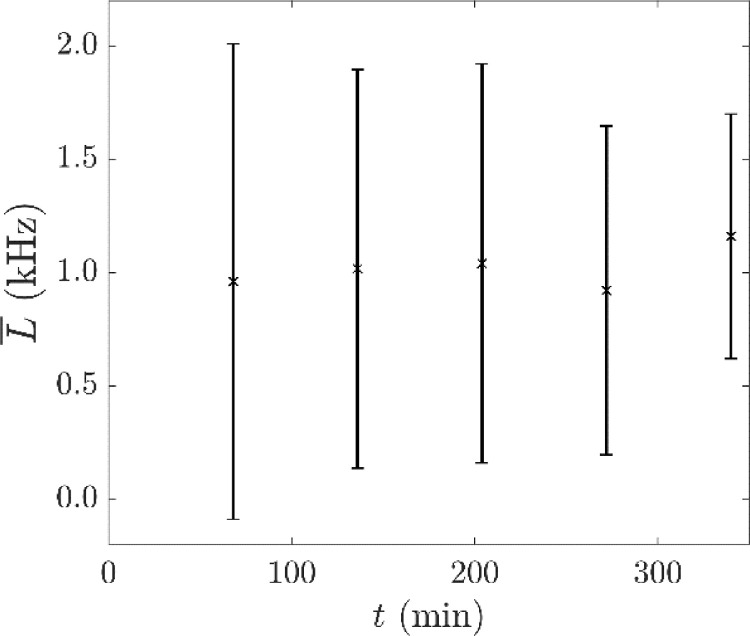
Average location rate $\bar{L}$ with time t of a random sample of 50 data points from the location data at each time with standard uncertainties of the mean $u(\bar{L})$.

The location rate, L, was calculated as the number of locations per second. A target location rate of 1 kHz was set, where the time interval between consecutive locations was approximately 1 ms to correspond to the 1 ms time stamp of the list mode data acquisition of the PET camera, which approaches the smallest increment of the time measurement. With the decay of the PET radionuclide, the number of lines of response recorded decreased with each 1 ms interval. To consistently produce location data with the target location rate, the N value was decreased with the activity of the tracer, as suggested by Chiti et al. [3]. The value of N was chosen to be the mean number of events recorded in each 1 ms interval. Fig. 5 shows this target bin size, $N^{\prime }$, as a function of the tracer activity, A, at a value of $f_{opt}$ = 0.30. The mean location frequency of a random sample of 50 locations from the data after locating the tracer particle with $N^{\prime }$ and $f_{opt}$ are shown in Fig. 6 which was consistent for the duration of the measurements.

**Fig. 7:**
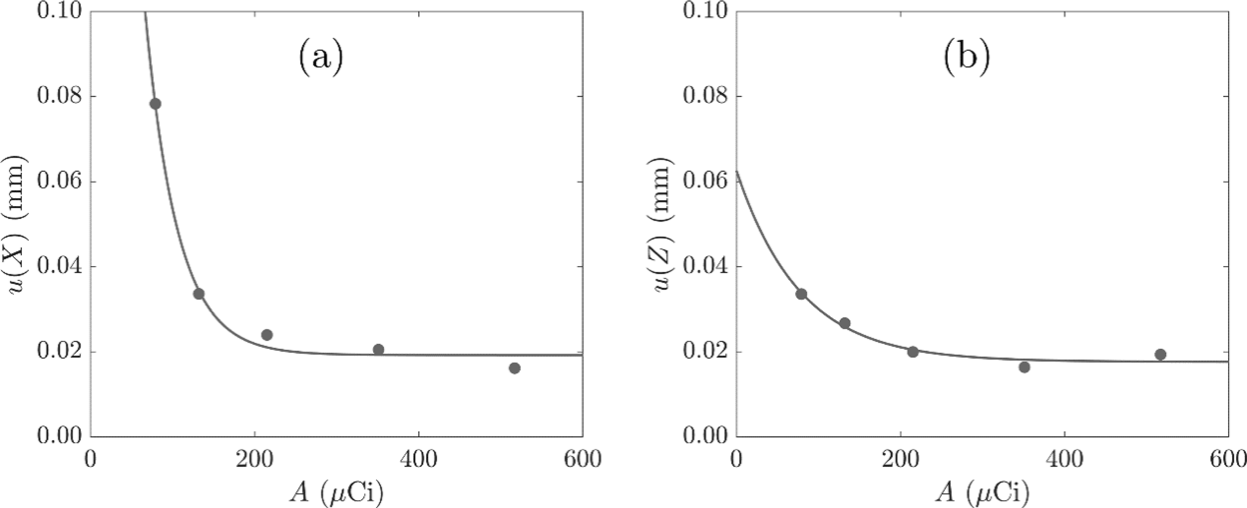
Uncertainty in the co-ordinates (a) X and (b) Z of the measured location against tracer activity A. The data were located with $N^{\prime }$ and f = 0.30 and show decay fits with *R*^2^ > 0.99.

Fig. 7 shows the location uncertainty with activity, calculated from the initial activity of ^68^Ga as a function of time, for locations derived with f = 0.30 and $N^{\prime }$ values from Fig. 5. The location uncertainty increases with decreasing activity of ^68^Ga and both u(X) and u(Z) tend to a minimum of 0.025 mm, which is considerably smaller than the size of the particle. The decay fit with high *R*^2^ value suggests that an increase in tracer activity beyond the maximum shown would not lead to a further decrease in the location uncertainty. The fitted decay relationships indicate a minimum activity of 150 to 200 µCi which corresponds to a minimum uncertainty in the location measurement in X and Z for arcs of radius 30 mm.

#### Methods to reduce noise in the location data

Smoothing was introduced to reduce high-frequency noise in the location data, using a cubic spline function as suggested by Cole et al. [4] with a compact kernel of finite height which is based on weights $w_{i}$,

(4) $$\begin{array}{c} w_{i}=1-6{q_{i}}^{2}+6{q_{i}}^{2}\;\;\;\;\text{for}\;\;\;\;q_{i}>0.5\;\text{and}\\ w_{i}=2{\left(1-q_{i}\right)}^{3}\;\;\;\;\text{for}\;\;\;\;0.5<q_{i}<1.0. \end{array}$$

The kernel $q_{i}$ is a function of the time of each location and weighting function width, which is equal to the half-kernel width Δt,

(5) $$q_{i}=\frac{\left|t_{i}-t\right|}{\Delta t}$$

The final PEPT co-ordinate $\hat{A}$ which corresponds to X, Y or Z, is calculated from the PEPT co-ordinate ${\hat{A}}_{i}$ at time i,

(6) $$\hat{A}=\frac{\sum w_{i}{\hat{A}}_{i}}{\sum w_{i}}.$$

**Fig. 8:**
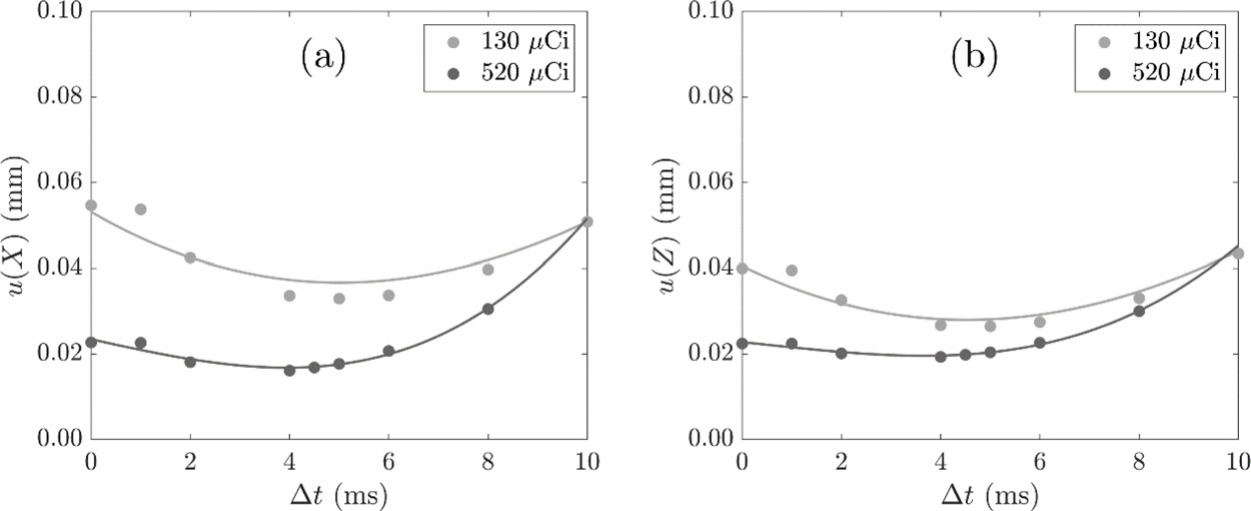
Uncertainty in the co-ordinates (a) X and (b) Z of the measured location with half kernel width Δt used in cubic spline smoothing for tracer activities 520 µCi and 130 µCi and located with $N^{\prime }$ and f = 0.30. Cubic fits with *R*^2^ > 0.9 are included.

Fig. 8 shows the uncertainty in the measured location with the half width of the kernel Δt. For both tracer activities considered, 520 µCi and 130 µCi, smoothing with small values of Δt of around 4 ms reduced the location uncertainty to its minimum value.

#### Methods to evaluate the standard uncertainty in the velocity data

The weighted average for the velocity was calculated over 11 adjacent positions as suggested in the six points method by Stewart et al. [15]. The predicted velocities $V_{fit,X}\left(t\right)$ and $V_{fit,Z}\left(t\right)$ in two co-ordinates were calculated from the optimum values of a, ν and ϕ found previously for each location procedure, where

(7) $$\begin{array}{c} V_{fit,X}\left(t\right)=-2\pi \nu a\sin\left(2\pi \nu t+\phi \right)\;\;\text{and}\\ V_{fit,Z}\left(t\right)=\;-2\pi \nu a\sin\left(2\pi \nu t+\phi +\frac{\pi }{2}\right). \end{array}$$

The root mean squared differences between the predicted and measured velocity in the X and Z co-ordinates were calculated as

(8) $$\begin{array}{c} \delta v_{X}=\;\sqrt{\frac{\sum_{i}^{n}{\left(\Delta v_{X,i}\right)}^{2}}{n}}\;\;\text{and}\\ \delta v_{Z}=\;\sqrt{\frac{\sum_{i}^{n}{\left(\Delta v_{Z,i}\right)}^{2}}{n}}\;, \end{array}$$

where $\Delta v_{X,i}=v_{X,i}-V_{fit,X}\left(t_{i}\right)$ and $\Delta v_{Z,i}=v_{Z,i}-V_{fit,z}\left(t_{i}\right)$ and the values of $v_{X,i}$ and $v_{Z,i}$ were calculated with the six points method [15] Eq. 9,

(9) $$\begin{array}{ccc} v_{X} & = & 0.1\left(\frac{X_{i+5}-X_{i}}{t_{i+5}-t_{i}}\right)+0.15\left(\frac{X_{i+4}-X_{i-1}}{t_{i+4}-t_{i-1}}\right)+0.25\left(\frac{X_{i+3}-X_{i-2}}{t_{i+3}-t_{i-2}}\right)+0.25\left(\frac{X_{i+2}-X_{i-3}}{t_{i+2}-t_{i-3}}\right)\\ & & +\;0.15\left(\frac{X_{i+1}-X_{i-4}}{tx_{i+1}-t_{i-4}}\right)+0.1\left(\frac{X_{i}-X_{i-5}}{t_{i}-t_{i-5}}\right),\\ v_{Z} & = & 0.1\left(\frac{Z_{i+5}-Z_{i}}{t_{i+5}-t_{i}}\right)+0.15\left(\frac{Z_{i+4}-Z_{i-1}}{t_{i+4}-t_{i-1}}\right)+0.25\left(\frac{Z_{i+3}-Z_{i-2}}{t_{i+3}-t_{i-2}}\right)+0.25\left(\frac{Z_{i+2}-Z_{i-3}}{t_{i+2}-t_{i-3}}\right)\\ & & +\;0.15\left(\frac{Z_{i+1}-Z_{i-4}}{t_{i+1}-t_{i-4}}\right)+0.1\left(\frac{Z_{i}-Z_{i-5}}{t_{i}-t_{i-5}}\right). \end{array}$$

The standard uncertainties in the velocity in each dimension were then calculated as

(10) $$\begin{array}{c} u\left(v_{X}\right)=\frac{\delta v_{X}}{\sqrt{n}}\;\;\text{and}\\ u\left(v_{Z}\right)=\frac{\delta v_{Z}}{\sqrt{n}}. \end{array}$$

**Fig. 9:**
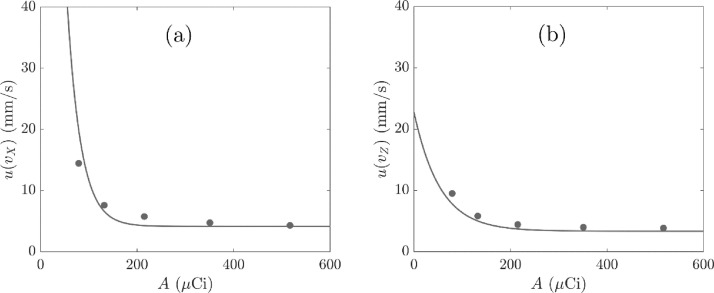
Uncertainty in the tracer velocity in the (a) X and (b) Z co-ordinates of measured tracer location against tracer activity, A. The data were located with $N^{\prime }$ and f = 0.30 and show decay fits with *R*^2^ > 0.99.

Fig. 9 shows the uncertainty in the velocity in X and Z. The results suggest a similar minimum activity of 150 to 200 µCi to achieve a minimum uncertainty in the measurement of the impeller tip speed.

**Fig. 10:**
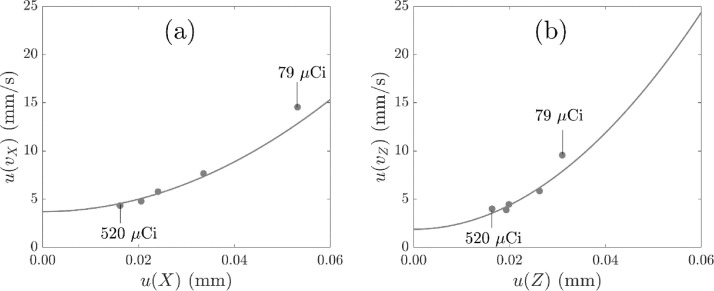
Uncertainty in the velocity against uncertainty in the co-ordinates (a) X and (b) Z of the measured tracer location with tracer activity and the data were located with $N^{\prime }$ and f = 0.30. Quadratic fits with *R*^2^ > 0.9 are included.

Fig. 10 shows the uncertainty in the velocity with the uncertainty in the location for two co-ordinates X and Z. A quadratic fit is included between the two types of uncertainty, in position and velocity, which suggests that the uncertainty in the velocity can be predicted from the uncertainty in the location of the tracer for the tracer activities included here, as proposed by García-Triñanes et al. [10].

### Protocol for locating freely moving tracer particles from PEPT measurements in the flotation vessel

**Fig. 11:**
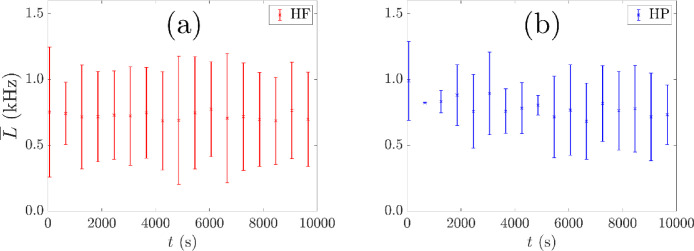
The average location frequency $\bar{L}$ with time for two experiments with different tracer particles (a) HF – hydrophilic and (b) HP – hydrophobic, together with standard uncertainties in the average location frequency $u(\bar{L})$ as calculated from a random sample of 50 points from each data file.

Based on the results of the rotating disk study, the parameters for locating freely moving tracer particles in the flotation vessel are f = 0.30 and a target bin size, $N^{\prime }$, which is related to the mean number of lines of response recorded per 1 ms time interval. The target bin size is also reduced linearly with tracer activity, A, relative to the activity at the start of each listmode file recording to maintain a location frequency of approximately 1 kHz as shown in Fig. 11 for two different PEPT experiments at consistent flotation conditions. The standard uncertainties are relatively large in magnitude (comparable to the order of magnitude of $\bar{L}$) because of the location method. By locating the particle with a fixed sample size of lines per location, the time interval spanned by each sample of lines varies with the attenuating properties of the local media, which change with the non-uniform mass density of the solid and gas suspension in the fluid, and the tracer velocity, which fluctuates with the turbulent properties of the fluid.

It should be noted that there is no "optimum" N for PEPT studies when using “*track”*, and varying the value impacts the location rate and uncertainty in the location measurement. The parameters for locating the tracer particle selected here are based on the motion of a tracer particle moving in a circle (radius of 30 mm) at constant velocity (3.7 m/s). This motion is not necessarily characteristic of the full range of motion of the tracer particle inside a flotation vessel and further research is required to develop a scheme that produces a sequence of locations that represent all features in the tracer motion such as arcs of different radii in turbulent vortices and high rates of acceleration. The main motivation for utilizing a location scheme related to the tracer activity is to facilitate the direct comparison of consecutive paths in any region of interest in the flotation vessel, following previous studies such as Yang et al. [19] which reported differences in location uncertainty from measurements of stationary tracer particles with different activities.

Following the rotating disc study, list-mode data recorded at activities lower than 150 µCi are not used to produce location data at a location rate of 1 kHz as these are correlated with an increase in uncertainty in location and velocity measurement. The location data are smoothed with a cubic spline kernel of half width of 4 ms to reduce noise in the location data.

**Fig. 12:**
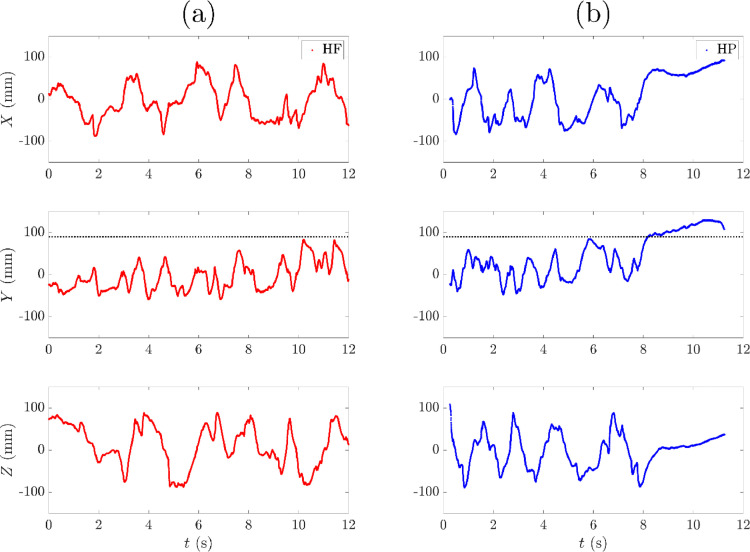
Locations of two tracer particles (a) HF – hydrophilic, (b) HP – hydrophobic in the co-ordinates X,Y, and Z with time, *t*, in repeat flotation experiments as located with the standard protocol. The dotted lines indicate the vertical position of the pulp-froth interface.

**Fig. 13:**
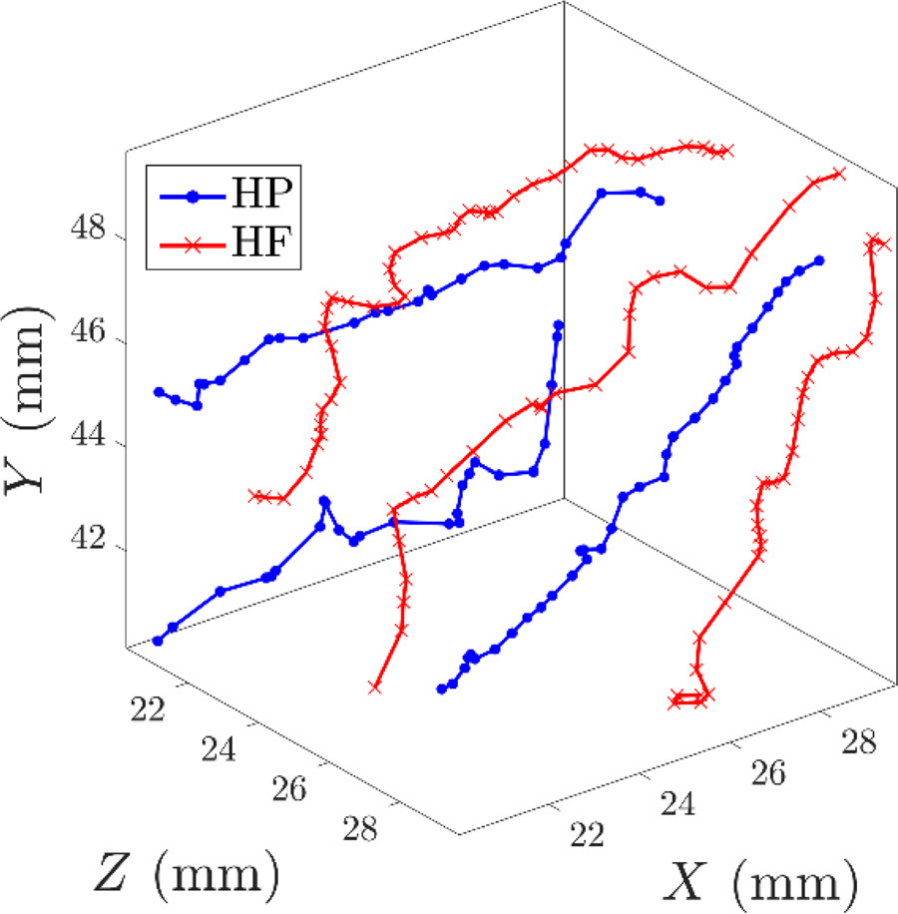
Example trajectories of two different tracer particles (HF – hydrophilic, HP – hydrophobic) in a 1 cm^3^ region of interest defined by the co-ordinates *X, Y* and *Z* in repeat flotation experiments with locations derived from the standard protocol.

Examples of the resulting trajectories of two freely moving tracer particles using this protocol are shown in Fig. 12 for a repeat flotation experiment. On this scale, the location data appear as continuous lines due to the high 1 kHz location rate. To visualize the location rate closer to the scale of the tracer particle, trajectories of two tracer particles are also represented in a 1 cm^3^ region of interest near the impeller in Fig. 13.

### Other co-ordinate systems

**Fig. 14:**
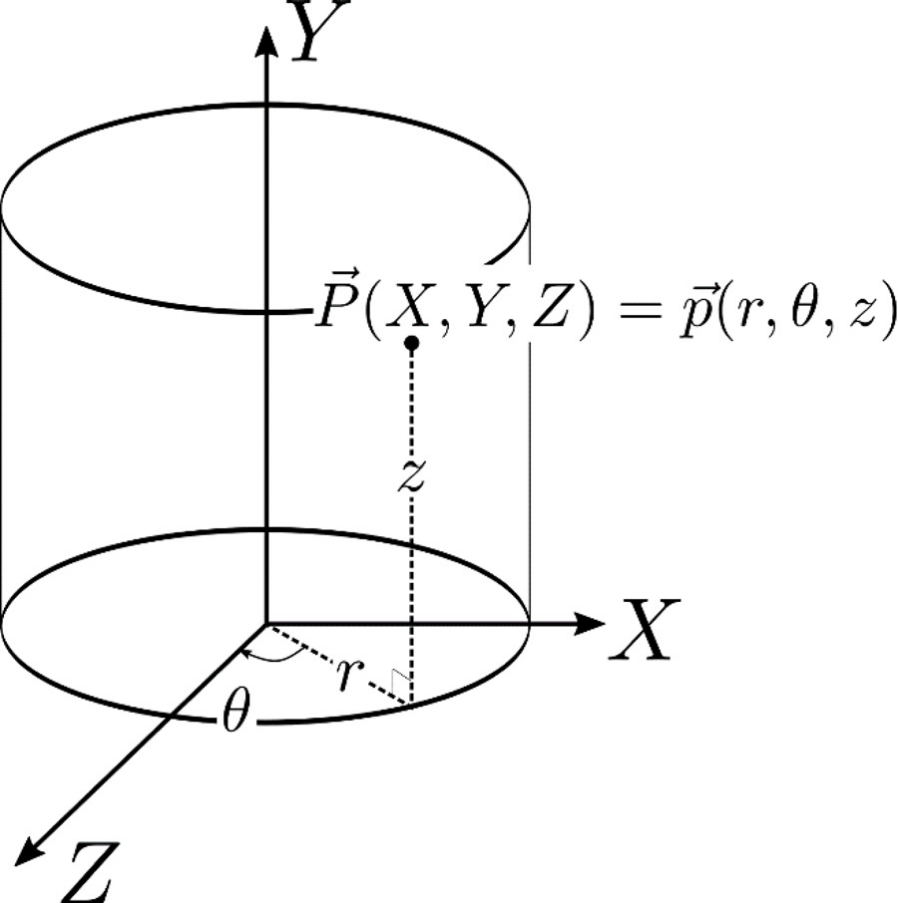
Tracer position in the Cartesian co-ordinate system, $\vec{P}\left(X,Y,Z\right)$, relative to the configuration of the field of view of the HR++ positron camera and the cylindrical polar co-ordinate system, $\vec{p}\left(r,\theta ,z\right)$, relative to the orientation of the flotation vessel.

The measured positions in Cartesian co-ordinates, $\vec{P}\left(X,Y,Z\right),$ are converted to cylindrical polar co-ordinates, $\vec{p}\left(r,\theta ,z\right)$, with time, t, where z corresponds to the axial length of the reactor vessel and is equivalent to the vertical component Y in $\vec{P}\left(X,Y,Z\right)$. The relationship between these two co-ordinate systems is illustrated in Fig. 14.

### Time averaging methods

**Fig. 15:**
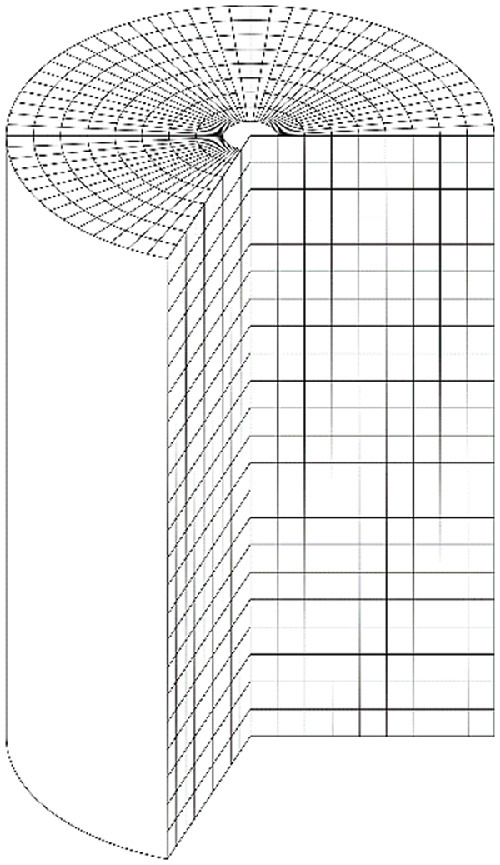
Schematic of the voxel scheme in cylindrical polar co-ordinates.

Around two hours of velocity data per tracer are used to create time averaged 3D Eulerian flow representations with voxel lengths from 2 to 10 mm. A voxel scheme based on Cartesian co-ordinates is used to calculate horizontal slices of the average tracer velocity with different vertical positions and are represented as average trajectories or streamlines. In this case the voxels are cubes and have equal volume. A voxel scheme in cylindrical polar co-ordinates is used to calculate horizontal and azimuthal slices of the distribution of average velocity component values to calculate streamline and quiver plots. In this case the volume of the voxels increases with radial position as shown in Fig. 15, and different angular spans can be used to measure average velocity behaviour at different angles around the circumference of the vessel. It is common practice to use a span of 5°.

**Fig. 16:**
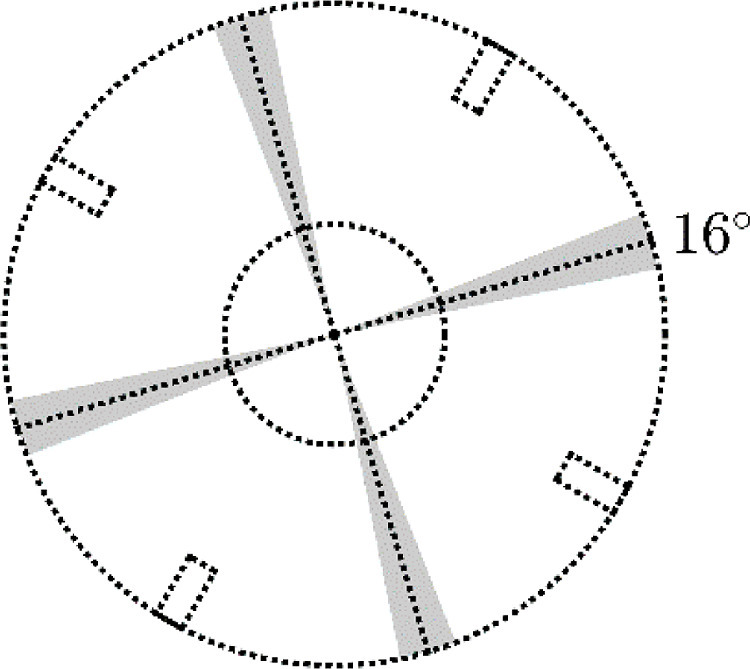
Horizontal cross-section of the vessel showing the four symmetrical azimuthal slices.

When using cylindrical polar co-ordinates, four slices of the data in equivalent azimuthal slices in the vessel are taken and summed to produce the time-averaged data arrays, starting at a particular angle plus increments of 90°, as shown for a starting angle of 16° in Fig. 16 as the mid-angular position between the baffles. This employs the symmetry of the vessel along the vertical dimension of the field of view of the PET camera and axial dimension of the vessel to increase the number of data points within each voxel.

For each voxel, aesthetic histograms of the velocity data are produced to highlight the underlying velocity distribution of each tracer using Doane's formula for the optimum number of bins, *k*_opt_ [8], where,

(11) $$k_{\text{opt}}=1+\log_{2}\left(n\right)+\log_{2}\left(1+\frac{\left|\gamma_{1}\right|}{\sigma_{\gamma_{1}}}\right)$$

(12) $$\text{and}\;\sigma_{\gamma_{1}}=\sqrt{\frac{6\left(n-2\right)}{\left(n+1\right)\left(n+3\right)}}.$$

Eq. 12 relates the number of data points, *n*, and the skewness of the distribution, *γ*_1_, as determined by the Fisher-Pearson coefficient of skewness which is based on the third standardised moment around the mean. The value of *k*_opt_ increases with *n* and with skewness to provide a greater number of bins and thus better resolution to closely reflect the underlying distribution in the data. After determining *k*_opt_ for each voxel, the bin width, *h*, was calculated between the maximum and minimum value of each velocity recorded for each voxel, using

(13) $$h=\frac{v_{\text{max}}-v_{\text{min}}}{k_{\text{opt}}}.$$

The modal average of the distribution of tracer velocity in each voxel is preferred to the mean average as it represents the peak velocity in distributions that are asymmetrical or multi-modal. For a more precise value of the peak tracer velocity in each voxel, the probability density function (PDF) of the distribution of all velocity values logged in that voxel is estimated with a Gaussian kernel and the maximum peak is found to the nearest 1 mm/s. Voxel data are excluded from the results when the distribution contains fewer than 25 data points, as this would be insufficient to velocity data to characterize the peak of the PDF of the distribution of velocity.

**Fig. 17:**
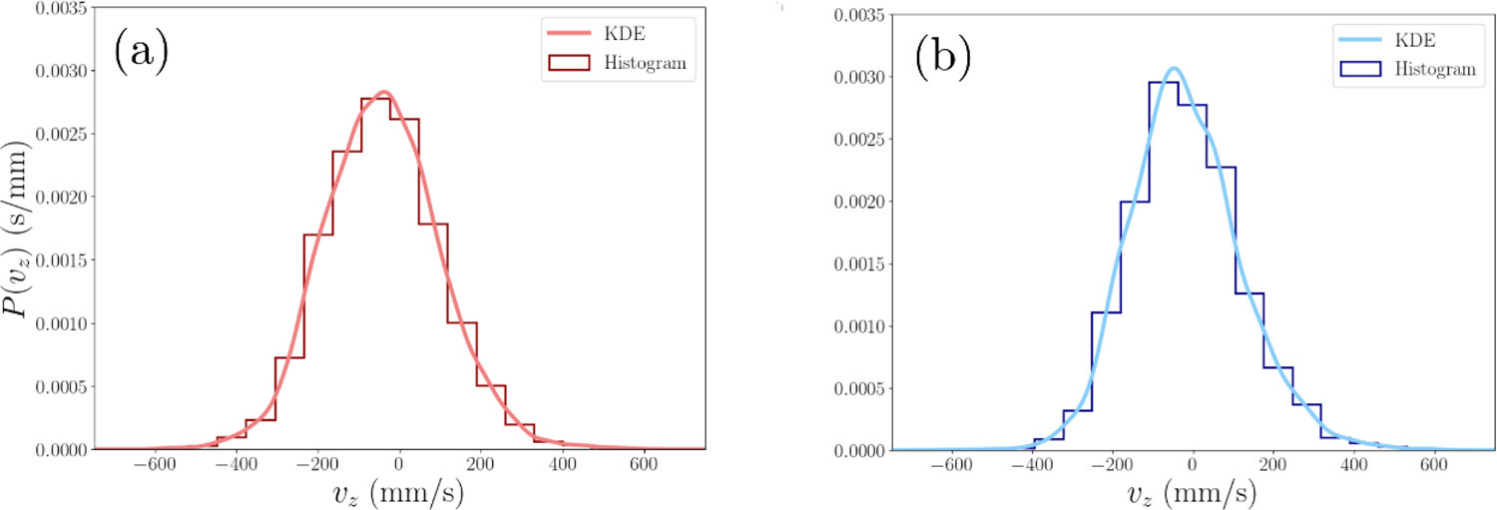
Probability density functions of the vertical velocity distribution $v_{z}$ of two tracer particles (a) hydrophilic and (b) hydrophobic in a 1 cm^3^ region of interest in the flotation vessel. The distributions are represented by two different styles: a kernel density estimate (KDE) and histogram with Doane's optimum bin width [8].

Fig. 17 shows a comparison of the two methods for representing the distribution of measured tracer velocities in a voxel, aesthetic histograms and kernel density estimates (KDE) of the PDF, where the voxel is a region of interest in the vessel to compare the behaviour of hydrophilic and hydrophobic tracer particles. Both methods can be used to find the peak of the distribution, with the KDE tending to find the peak to the nearest 1 mm/s and the histogram to a precision of the bin width.

**Fig. 18:**
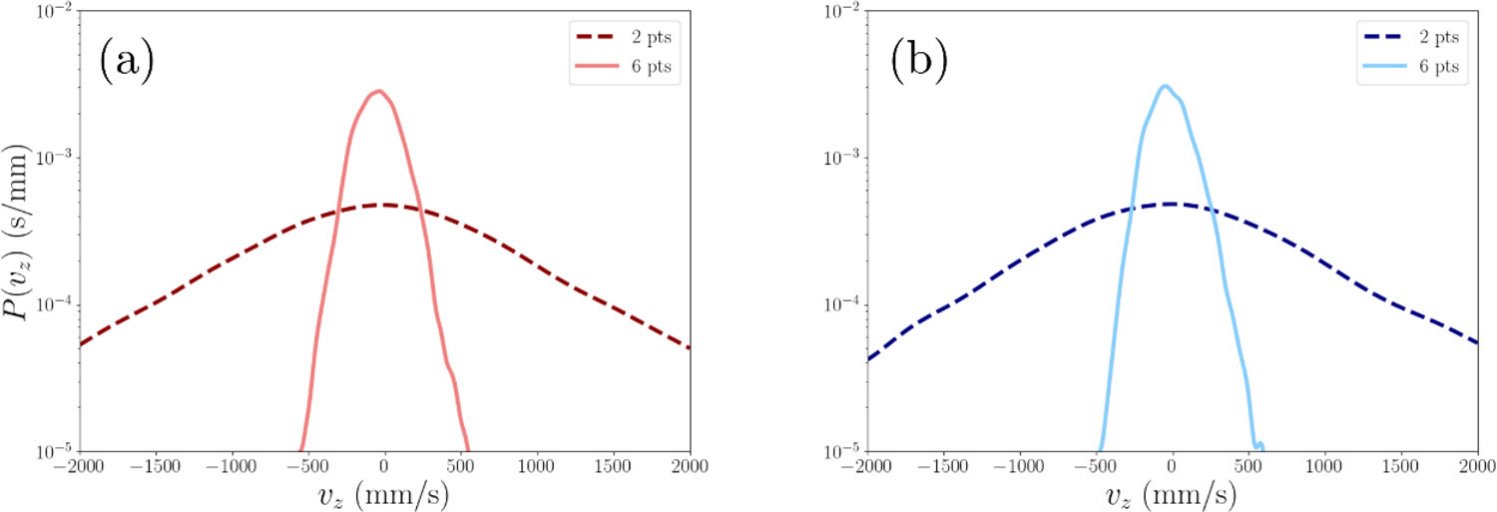
Probability density functions of the vertical velocity distribution $v_{z}$ of two tracer particles (a) hydrophilic and (b) hydrophobic in a 1 cm^3^ region of interest in the flotation vessel. Kernel density estimates from two different velocity treatments are shown: “6 pts” from Stewart et al. [15] and “2 pts” is a 1st order forward finite difference scheme.

The PDFs of tracer velocity can be used to illustrate the impact of using the six points velocity calculation [15], which tends to reduce noise in the velocity data propagated from the 3D position and time measurements of the location data. As shown in Fig. 18, the noise can be considerable when calculated with a first order finite difference scheme as represented by the width of the probability density function (PDF) of the velocity. This leads to extreme values in the PDF, in comparison to the narrower PDF for the six points methods. The six points method is widely used in PEPT for measurements of the average flow behaviour, however the compromise with this method is that it also smooths out turbulent features in the flow.

**Fig. 19:**
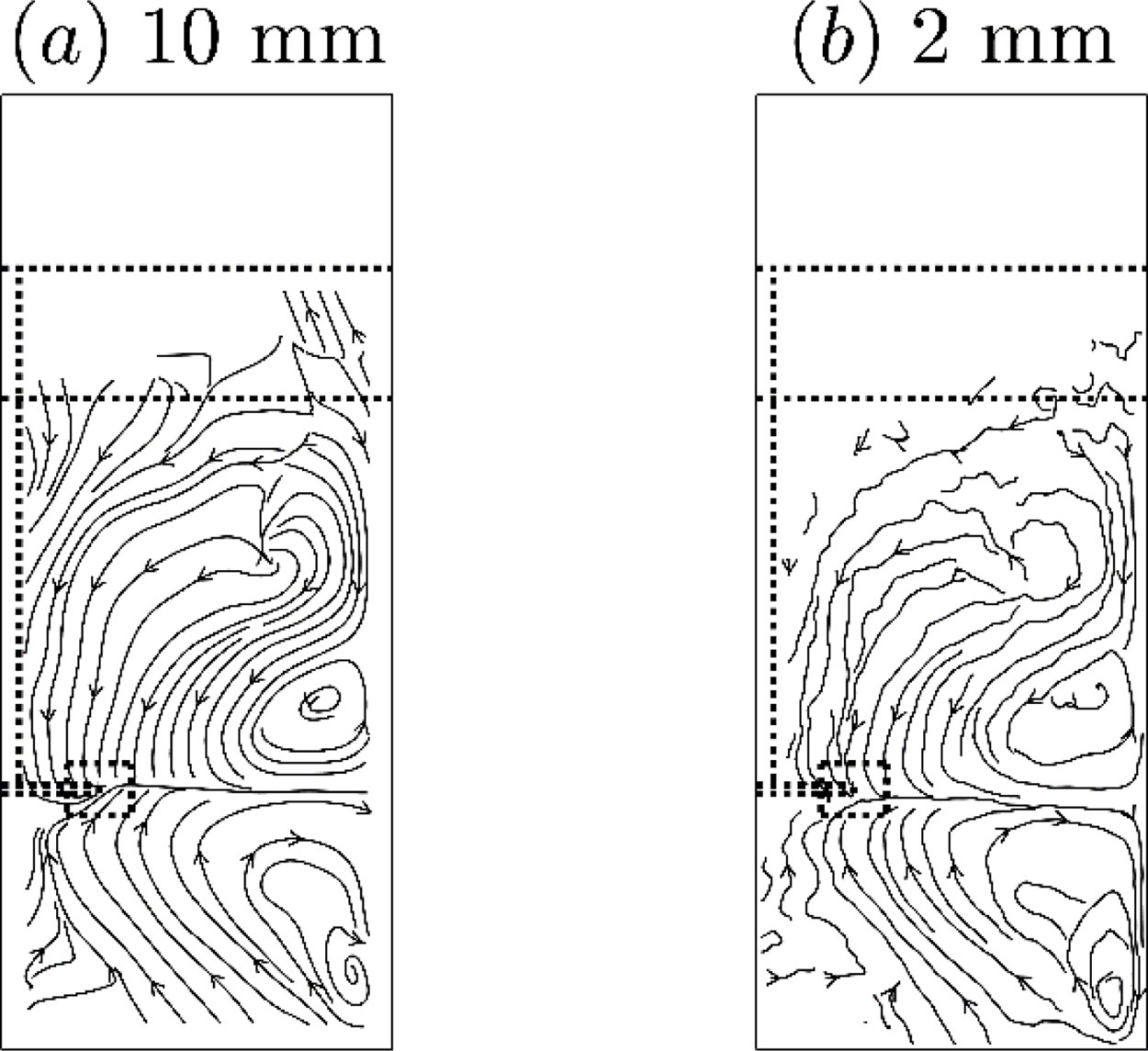
Average trajectory plots of azimuthal slices of the flow of a hydrophilic tracer in the flotation vessel as calculated with (a) 10 mm and (b) 2 mm voxel schemes from the same data. The impeller position is shown on the left and the interface and lip levels as horizontal lines.

Fig. 19 shows two representations of the streamlines or average trajectories of the average velocity of a hydrophilic tracer particle in an azimuthal slice, which were both derived from the same location data with two different voxel schemes. The streamlines in the 10 mm scheme are smoother and generally continuous, in comparison to the streamlines in the 2 mm scheme which show higher noise levels towards the froth. Therefore, in the standard method, streamlines are plotted for voxel schemes with 10 mm length.

**Fig. 20:**
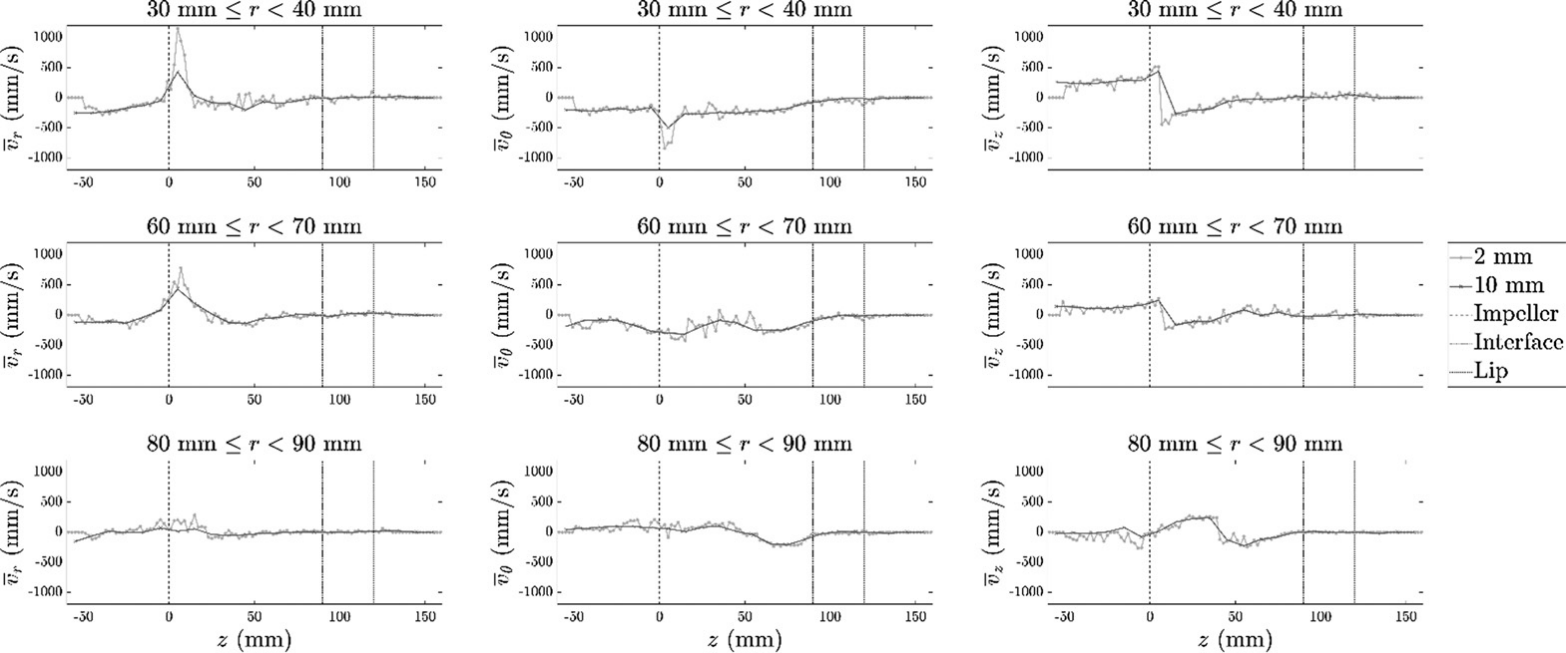
Vertical profiles of the modal average velocity behaviour of the hydrophobic tracer with height of the vessel (z) for the radial velocity ${\bar{v}}_{r},\;$tangential velocity ${\bar{v}}_{\theta }$ and vertical velocity ${\bar{v}}_{z}$. The velocity data for different radial positions, r are shown for both 10 mm (×) and 2 mm (°) voxel schemes. The impeller tip speed was 3700 mm/s. The levels of features of the vessel are shown at different constant z values for reference to the flotation vessel geometry including z=0mm for the impeller, z=90mm for the interface and z=120mm for the lip.

Both voxel schemes tend to the same average velocity behaviour for each component as shown in Fig. 20 for a series of vertical profiles with different radius in the vessel. The profiles from the larger 10 mm voxel length show fewer small-scale fluctuations in velocity and tend to underestimate the maximum and minimum velocity values in comparison to the 2 mm voxel length. The profiles from the 2 mm voxel data correspond to a subset (1/25) of the 10 mm voxel data.

**Fig. 21:**
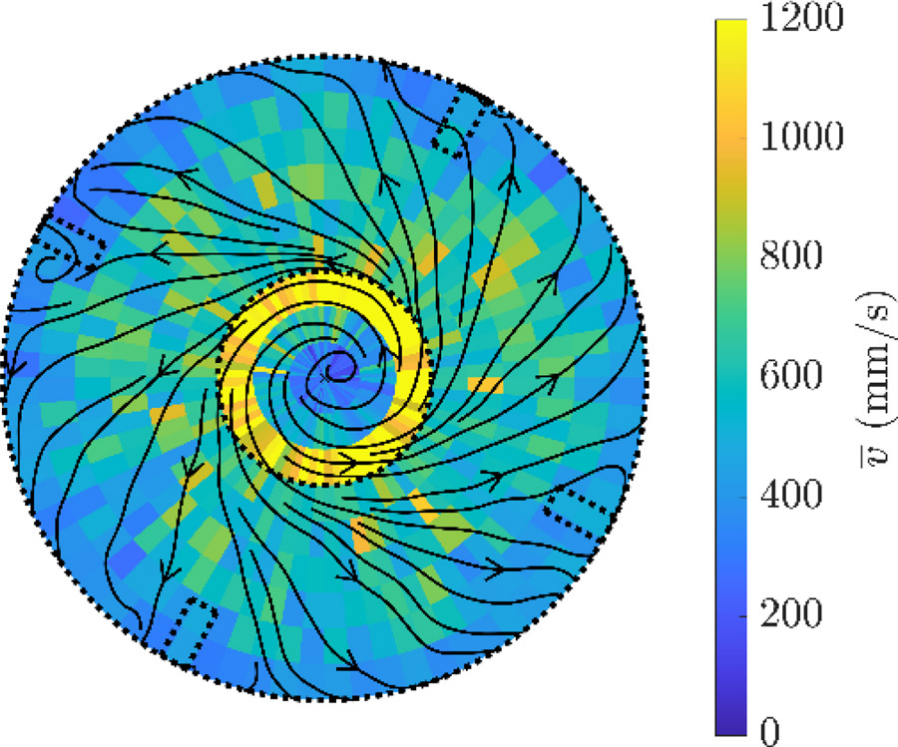
Horizontal slice through the PEPT measurement of the velocity of a hydrophobic tracer particle at the height of the impeller, y = 0 mm, showing the average velocity, $\bar{v}$, in a cylindrical polar co-ordinate scheme and the average flow streamlines.

An example of a horizontal 2D slice through the data in shown in Fig. 21 for a voxel length of 10 mm and span 5°.

**Fig. 22:**
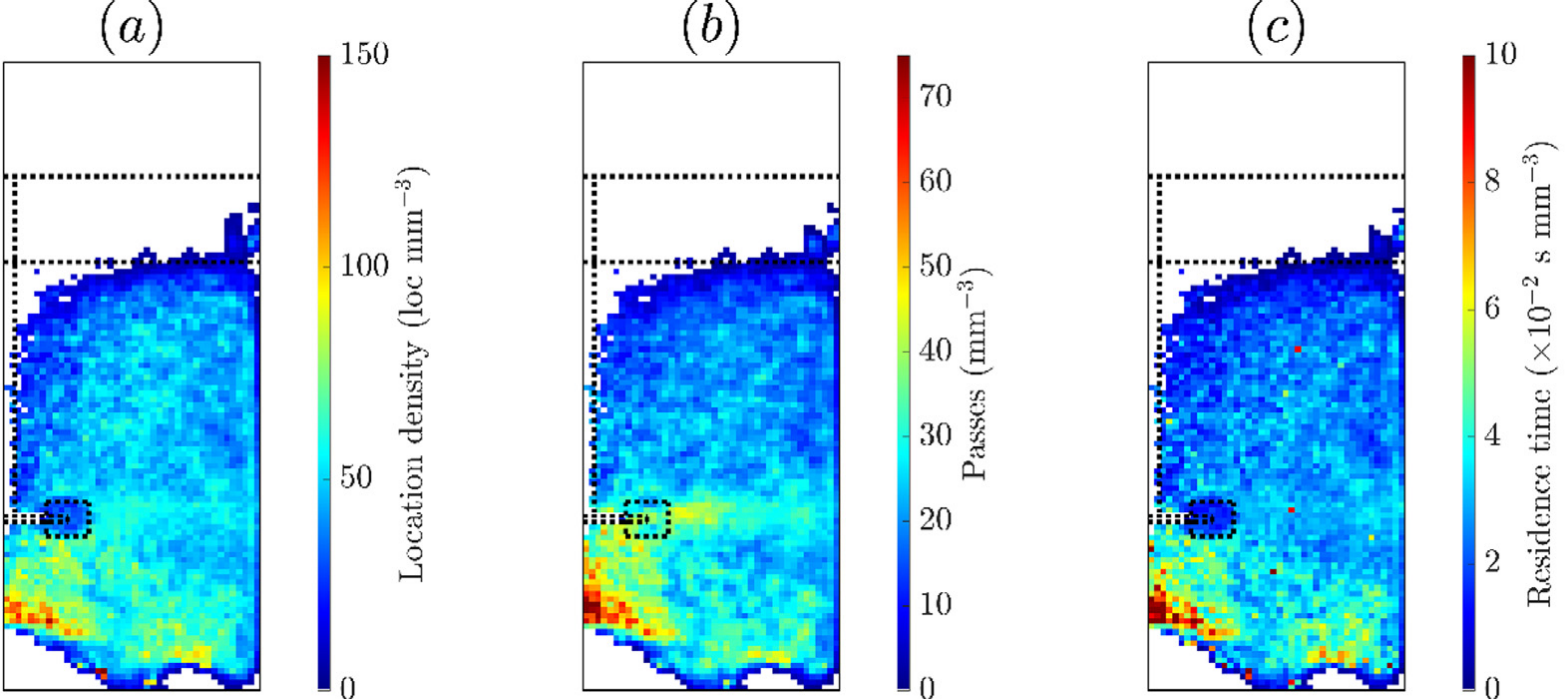
Different representations of occupancy for azimuthal slices of the motion of a hydrophilic tracer particle in the flotation vessel: (a) location density, (b) number of passes per volume and (c) residence time per volume. The impeller position is shown on the left and the interface and lip levels as horizontal lines.

The tracer occupancy is represented in three ways: the location density as described by the number of locations per volume, the number of passes of each volume and the residence time of the tracer per volume. Examples of these representations are shown in Fig. 22, with each normalized by the varying voxel volume. The three representations are similar with highest occupancy underneath the impeller which is also above the air plate. The biggest differences occur around the impeller blade and in the discharge plane. The location density is low near the impeller blades and higher in the discharge stream. The number of passes is high near the impeller and in the discharge plane, whereas the residence time is low both near the impeller blades and discharge stream. The location rate is not constant in the flotation data when using a fixed number of lines of response with the “*track”* algorithm. It is velocity dependent, with a lower rate in regions of high speed, which may lead to misrepresentations of occupancy with location density in regions with high acceleration as shown in Fig. 22 (a). The number of passes of each voxel is shown in Fig. 22 (b) and shows the tracer visits the voxels near the impeller with high frequency. The residence time near the impeller is also low in Fig. 22 (c), which is also related to the high tracer speed in that region.

When comparing occupancy across different experiments, the representations are normalized by the total occupancy of the slice or of the vessel to ensure equivalent sample size between experiments. The tracer may get “stuck” with implication for the total occupancy count, be it time, locations or passes. As an example, hydrophobic tracer particles can get stuck in the stagnant region of the froth in the middle of the vessel and the corresponding occupancy data may need to be removed from the total to avoid skewing the occupancy elsewhere in the vessel.

## Conclusions

This work presents a standard operating protocol for PEPT measurements in a froth flotation vessel with the HR++ camera at PEPT Cape Town in South Africa. Measurements are performed at consistent flotation conditions as determined by the air recovery, to ensure the behaviour of tracer particles with different properties can be directly compared. A protocol for locating tracer particles was developed from a rotating disk study which locates tracer particles relative to activity. Voxel schemes in Cartesian co-ordinates and cylindrical polar co-ordinates are used for time averaged analyses, to describe the average flow behaviour in the flotation vessel in azimuthal and horizontal slices. The average tracer velocity values are determined from the peak of the PDF of the distribution of velocity values in each voxel as estimated with a kernel density approach, to represent the most probable velocity value in all shapes of distribution, including asymmetrical and multimodal distributions.

This protocol enables the wider application of PEPT to investigations of particle behaviour in coarse particle flotation, including the effect of different particle properties, flotation conditions and vessel design.
